# Automatic Grasping System and Hybrid Controller Towards Multi-Drone Parcel Delivery

**DOI:** 10.3390/s26020653

**Published:** 2026-01-18

**Authors:** Bruno J. Guerreiro, Francisco Azevedo, Paulo Oliveira, Rita Cunha

**Affiliations:** 1 DEEC/CTS/LASI, NOVA School of Science and Technology, Universidade NOVA de Lisboa, 2829-516 Caparica, Portugal; 2 ISR/LARSYS, Instituto Superior Técnico, Universidade de Lisboa, 1049-001 Lisboa, Portugal; francisco.azevedo@tecnico.ulisboa.pt (F.A.); paulo.j.oliveira@tecnico.ulisboa.pt (P.O.); rita.cunha@tecnico.ulisboa.pt (R.C.); 3 LAETA and DEM, Instituto Superior Técnico, Universidade de Lisboa, 1049-001 Lisboa, Portugal

**Keywords:** hybrid MPC, pose estimation, drone delivery, gripper, 3D printing

## Abstract

This paper presents the development of an autonomous grasping mechanism for drone-based parcel delivery systems towards developing capabilities for in-flight package transfer. The approach integrates a mechanical gripper fitted with sensors and a pose estimation method for parcels, all coordinated through a hybrid Model Predictive Control (MPC) architecture. The gripper’s mechanical structure and prototype are developed using 3D printing technology for both the main framework and gear components. A hybrid dynamical model is formulated that integrates the gripper mechanics with simplified drone dynamics, capturing distinct operational phases including package acquisition, transport, and release. The hybrid MPC framework computes reference trajectories for both the gripper arm configuration and the drone’s spatial path toward designated target positions. Experimental validation is conducted using the operational gripper prototype and pose estimation system, while drone behavior is represented through simulation.

## 1. Introduction

In the early stages of unmanned aerial vehicle (UAV) adoption, commonly known as drones, the dominant applications centered on observation and surveillance tasks. However, contemporary technological advances and growing demands for automation and remote operation have expanded the scope of UAV capabilities. Equipping these platforms with manipulation functionality enables not merely environmental observation but active physical interaction with surroundings as well. The inherent mobility of UAVs can increase the potential for ubiquitous object grasping and transportation, eliminating requirements for elaborate ground-based infrastructure. Addressing these opportunities, the REPLACE project [1] pursues the development of a rapid parcel delivery system for urban settings using drone platforms, acknowledging that relay operations between multiple drones become essential when individual vehicle range and endurance prove insufficient for complete delivery missions.

This paper focuses on developing a lightweight, fast, and autonomous grasping system for drone-based parcel exchange operations. The primary objectives are as follows: (i) a gripper design capable of handling parcels with known characteristics under constraints of mass, velocity, and energy consumption; (ii) establishing a perception and control architecture for the grasping device that enables package pose detection and responsive actuation. This foundation work can afterwords support the creation of basic grasping agents suitable for executing parcel transfers in operational contexts.

The task of aerial package manipulation and transport presents challenges that span a spectrum of scenarios with varying complexity levels. At the simplest level, a vehicle retrieves a package from one stationary ground location and deposits it at another fixed position. Upon approaching the pickup zone, the gripper system engages and computes an optimal approach maneuver and path based on the detected package position. Once the payload is secured, the vehicle navigates to the delivery site for package release. A more demanding scenario involves acquiring objects from mobile platforms (such as another aerial vehicle or ground vehicle), where the cargo remains visible and accessible from above. Advanced scenarios might integrate the motion of both pickup and delivery platforms within a supervisory control strategy to optimize transfer speed and efficiency, such as the work published in [2,3].

Concerning gripper system design, ref. [4] provides an extensive kinematic analysis of 64 linkage-based gripper configurations, establishing a foundation for subsequent investigations. The work in [5] documents established industrial practices and design methodologies for gripping mechanisms, providing foundational principles for the present study. Additionally, ref. [6] addresses the integration of grasping mechanisms with aerial platforms, emphasizing vehicle dynamics and control architecture, whereas [7,8] explore the deployment of both impactive and ingressive gripper types on UAV systems. Comprehensive reviews of aerial manipulation technologies and methodologies are presented in [9,10,11], which discuss various gripper designs, control strategies, and application scenarios.

For grasping operations in uncertain or incompletely characterized environments, such as aerial load transport, perception and sensing capabilities become critically important. The research presented in [12] addresses this topic by examining UAV modeling and control with explicit consideration of environmental interactions. The work in [13] develops a vision-based control approach for quadrotor perching maneuvers on cables, though the challenge intensifies when accurate pose estimation of target objects becomes necessary, for which the methodology presented in [14] provides an effective and widely adopted solution. Recent developments include sophisticated rigid designs such as the dual-arm aerial manipulator with anthropomorphic grippers [15] or lightweight adaptive gripper for parcel delivery [16]. While rigid grippers offer high load capacity and predictable behavior, soft and soft–rigid hybrid designs provide compliant grasping with improved adaptability to geometric uncertainty, as demonstrated in [17] for food handling and in [18] with variable stiffness grippers. Specifically for the aerial parcel delivery application, ref. [19] provides an thorough review of the state-of-the-art technologies and challenges, highlighting the trade-offs between different gripper designs. The approach presented here emphasizes a streamlined simple grasping device combined with coordinated autonomous control of both vehicle and manipulation subsystems for package exchange operations.

Problems featuring multiple operational phases with both continuous and discrete state dynamics may require hybrid dynamical modeling approaches. The foundational concept of hybrid continuous-time dynamical systems was established in [20], which examined how discrete state variables can capture transitions among continuous dynamics modes and their stability properties. The hybrid automaton framework, introduced in [21] and elaborated in [22], provides a formal representation for hybrid systems, though numerous alternative formulations appear throughout the literature, such as [23]. The work in [24] advances toward more tractable frameworks for describing, analyzing, implementing, and controlling such systems through a Mixed Logical Dynamical (MLD) formulation.

The main contributions of this paper are the design, implementation and experimental validation of a new automatic gripping system towards multi-drone parcel delivery comprising the mechanical and electronics systems, a pose estimation method, and a hybrid MPC strategy to achieve automatic planning and control for grasping parcels. The proposed hybrid MPC strategy is capable of coordinating both the drone motion and the gripper actuation through different discrete operation phases, including approach, grasping, and release maneuvers, alowing for the operation design to focus on goals and respective combination of cost functionals and contrainst, rather than using predefined trajectories that might not be able to cope with a moving parcel. The control algorithm is validated in simulation and an experimental validation trials for the gripper system is also presented.

The paper is organized as follows. Section 2 outlines the gripper mechanism design procedure, while Section 3 presents a package pose estimation method based on ArUco fiducial markers. Section 4 develops the dynamical models for both drone and gripper subsystems, subsequently integrating them into a unified hybrid model with an associated hybrid MPC controller. Section 5 describes the implemented prototype along with validation experiments that integrate the various system components, and Section 6 provides concluding observations and directions for future investigation.

## 2. Gripper Design

Gripping mechanisms represent well-established devices that exhibit diverse configurations depending on their intended application, operational constraints, and available fabrication materials. The objective here is not to present a universal gripper design methodology, but rather to document the development process for a mechanism tailored to the specific requirements of this application.

### 2.1. Motion Constraints and Prehension

The transported item (or its enclosure) is assumed to possess a rectangular prismatic geometry with uniform mass distribution, which given the vehicle’s payload capacity limitations, is constrained to remain below 500 g. For the fundamental scenario where the vehicle acquires and releases cargo at fixed ground locations, the gripper’s motion envelope is bounded only by the vehicle frame and ground surface. The gripper arm dimensions must respect both the vertical clearance when in the closed configuration and the lateral clearance required for the fully opened state. A slow prehension sequence would necessitate reducing vehicle speed such that arrival at the target position coincides with jaw closure completion. Following package acquisition, the gripper must retain secure hold throughout the transport trajectory to the destination.

To handle packages of varying dimensions and aspect ratios, the gripper jaw surfaces should maintain parallel orientation throughout their motion.Unlike angular jaw configurations, parallel jaw motion enables grasping from any accessible package surface, providing versatility across multiple acquisition scenarios while ensuring greater tolerance for positioning errors. Gripper mechanisms can be categorized into two primary types: ingressive and impactive. The present work focuses exclusively on the latter category, which operates through a straightforward principle where grasping is accomplished and sustained by normal forces applied by the jaw surfaces against opposing faces of the target object. Package retention results from the friction force $F_{f}$ generated at these contact interfaces. Selecting appropriate jaw surface materials that yield suitable friction coefficients $\mu_{s}$ when paired with the package surface is essential. For cardboard–rubber material pairs, $\mu_{s}$ typically ranges from 0.5 to 0.8.

### 2.2. Typical Forces During Operation

**Figure 1:**
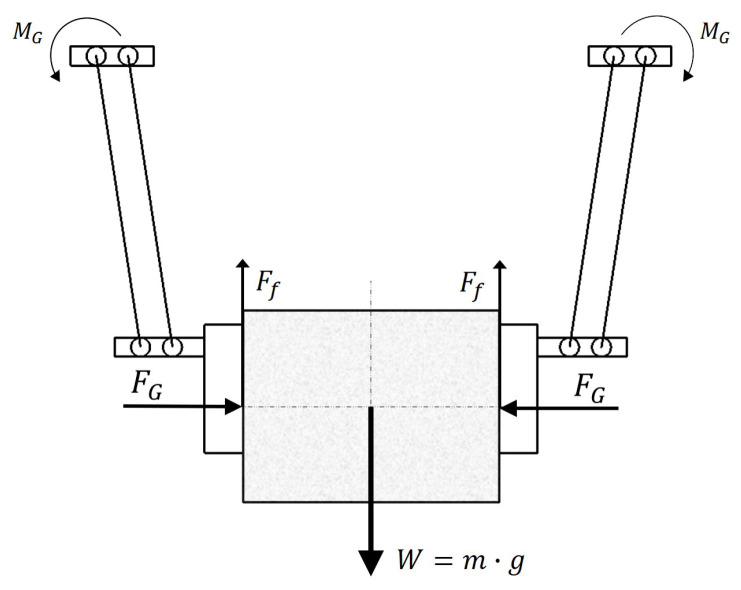
Forces acting on the object.

Developing an appropriate gripper mechanism requires identifying and quantifying the operational forces involved. Given the assumed cuboid package geometry, the contact region between the package and jaw surfaces forms a rectangular area. Larger contact areas enhance retention stability while simultaneously reducing required gripping forces. The adopted configuration employs symmetric bilateral grasping as illustrated in Figure 1.

Each jaw applies a normal force $F_{G}$ normal to the grasped package surface, whereas the friction force $F_{f}$ required to prevent the package descent is expressed as $F_{f}=\frac{m\;g}{n}$, where *n* denotes the number of contact points (fingers and jaws, in this case, 2), *m* represents the package mass, and *g* is the gravitational acceleration. Given the relationship $F_{f}=\mu_{s}N$, where *N* represents the normal contact force, the friction force $F_{f}$ and gripping force $F_{G}$ are related through $F_{G}=\frac{F_{f}}{\mu_{s}}$.

During package transport, the vehicle may undergo accelerations beyond gravitational effects in the vertical direction. The most demanding condition arises during rapid ascent maneuvers, when the vehicle experiences maximum acceleration $a_{max}$, which compounds the gravitational acceleration. Under these circumstances, considering a gripper arm length *l*, the forces and moments required to maintain secure package retention are, respectively,

(1) $$F_{G}^{*}=\frac{m\;\cdot \;(g+a_{max})}{\mu_{s}},$$

(2) $$M_{G}^{*}=F_{G}^{*}\;l.$$

### 2.3. Power Drive Chain

**Figure 2:**
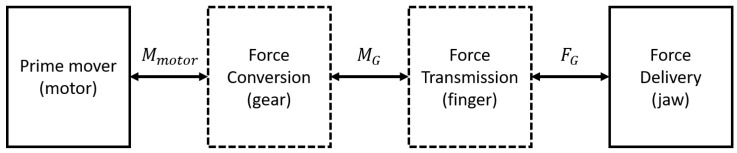
Structure of the gripper forces drive chain.

Selecting appropriate power transmission components is critical to meeting the system’s minimum velocity, force, and torque specifications. As illustrated in Figure 2, an actuator, specifically an electric motor, supplies torque $M_{motor}$ to the system.

Power transmission from the motor to the gripper finger rotation axis occurs through gear mechanisms, for which spur gears represent the most prevalent and elementary gear type. For a spur gear featuring *N* teeth, several fundamental geometric parameters can be established. The pitch circle defines a theoretical reference circle forming the basis for geometric calculations. The circular pitch, *p*, represents the arc length between corresponding points on adjacent teeth, measured along the pitch circle. The pressure angle α characterizes the inclination of the gear tooth profile. The module *m* serves as the standard ISO parameter for gear tooth sizing in gear nomenclature, defined as m=p/π, whereas the pitch diameter $d_{p}$ relates to the module through $d_{p}=Nm$.

For successful meshing between two spur gears, three principal requirements must be satisfied:

1. The gears must be mounted on parallel shafts;
2. Both gears must share identical module values *m*;
3. The shaft separation, the center distance, must equal half the sum of the two pitch diameters.

For two properly meshed gears *X* and *Y*, their gear ratio is expressed as

(3) $$S_{X-Y}=\frac{N_{Y}}{N_{X}}=\frac{d_{Y}}{d_{X}}.$$

During meshing operation, the pitch circles of both gears roll without slip, and the velocity at contact point *c* remains constant for gears with pitch radii $r_{X}$, $r_{Y}$, as well as angular velocities $\omega_{X}$, $\omega_{Y}$. Consequently, the angular velocities satisfy the relationship

(4) $$\frac{\omega_{X}}{\omega_{Y}}=\frac{r_{Y}}{r_{X}}=\frac{d_{Y}}{d_{X}}=S_{X-Y}.$$

Given that properly designed gear meshes exhibit high efficiency with approximately 2% losses, power transmission through the mesh is treated as constant.

The torques on each gear can be determined by equating the mechanical power transmitted, resulting in

(5) $$\frac{T_{X}}{T_{Y}}=\frac{\omega_{Y}}{\omega_{X}}=\frac{1}{S_{X-Y}}.$$

As such, the relationship between motor torque and the torque at the finger rotation axis is expressed as

(6) $$M_{G}=\frac{M_{motor}\;S_{total}}{n},$$

where $S_{total}$ represents the overall gear ratio of the complete transmission train (accounting for multiple gear stages if present).

**Figure 3:**
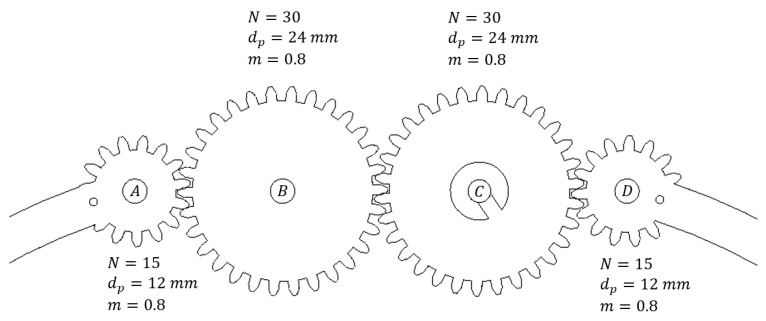
Gear train in one pair of gripper arms.

The grasping system comprises two pairs of gripper arms, with all elements designed to operate symmetrically and in synchronization, as depicted in Figure 3 for a single arm pair. Gears *B* and *C* maintain a 1:1 ratio, $S_{B-C}$, to preserve symmetry within each arm pair, while the ratios $S_{A-B}$ and $S_{D-C}$ are identical, and gear *B* functions as an idler, reversing the rotational direction of gear *A*.

Power transfer from the motor to the gear drive shaft employs a worm gear mechanism, which incorporates two components: a worm screw and a worm wheel (or worm gear). The transmission ratio for a worm drive is given by

(7) $$S_{worm}=\frac{N_{G}}{N_{W}},$$

where $N_{G}$ denotes the tooth count on the worm wheel and $N_{W}$ represents the number of thread starts on the worm screw. A key advantage of worm drives is their potential for self-locking behavior in certain configurations, where the worm wheel cannot back-drive the worm screw. Finally, combining Equations (1), (2) and (6) yields the minimum permissible total gear train ratio threshold, expressed as

(8) $$S^{*}=\frac{M_{g}^{*}}{M_{motor}}.$$

### 2.4. Final Prototype and Experimental Assessment

**Figure 4:**
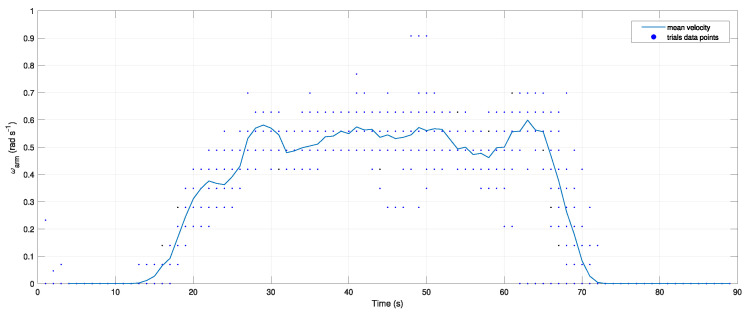
Mean gripper arm angular velocity measured from 30 different trials with the same step input reference.

The final gripper design was subject to several tests to validate its performance against the specified requirements. Regarding the use of the worm gear, from (4) and (5), it is possible to calculate the expected values of the gripper jaw’s maximum angular velocity and torque provided by the chosen motor and gear set combination, which are 5.39 Nm and 0.94 rad/s, respectively. However, empirical tests that account for losses and force transmission inefficiencies were performed for the angular velocity, as depicted in Figure 4.

The arm angular velocity was measured in 30 trials with the same step input reference, and the mean value was computed for each time step. From this data, it can be inferred that the maximum measured angular speed is ${\bar{\omega }}_{arm}\approx 0.6$ rad/s, which is about 64% of the expected values. Assuming the same losses for the available torque, $M_{motor}=3.45$ Nm. From (1) and (2), the minimum torque necessary to hold a parcel with m=200 g, considering $\mu_{s}=0.8$ and a combined maximum allowable acceleration of $12\;{\mathrm{ms}}^{-2}$, is $M_{G}^{*}=3$ Nm, which is within the estimated gripper capabilities, as $M_{motor}\geq M_{G}^{*}$.

**Figure 5:**
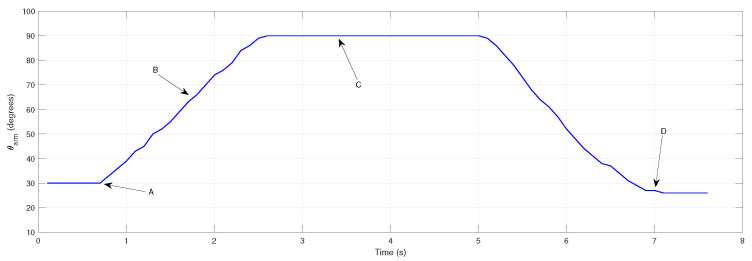
Evolution of the gripper arm angle $\theta_{arm}$, from fully open (A), closing (B), fully closed (C), and back to fully open (D).

To determine the gripper’s current angular position, a potentiometer is attached to one of the gripper arms. Experimental trials were performed to test the gripper going from fully open to fully closed, as shown in Figure 5, where the gripper arm angle $\theta_{arm}$ is plotted against time.

**Figure 6:**
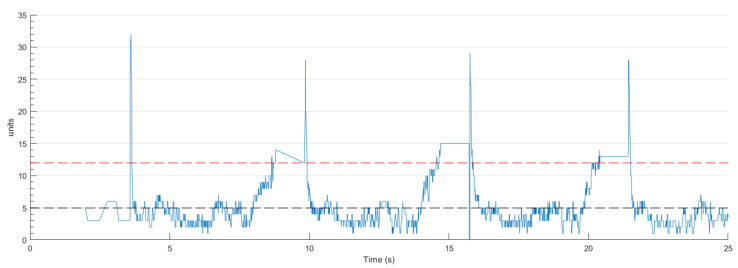
Current sensor circuit diagram. The black dashed line indicates the median current value, whereas the red dashed line represents the threshold established through a calibration procedure.

In addition to position tracking, the system must also verify whether the package has been successfully secured. This is accomplished by identifying instances when the servo motor stalls or experiences exceptional loading, indicating insufficient power to overcome mechanical resistance from an obstacle. A current detection circuit interfaced with the microcontroller was developed to acquire this data, as illustrated in Figure 6.

**Figure 7:**
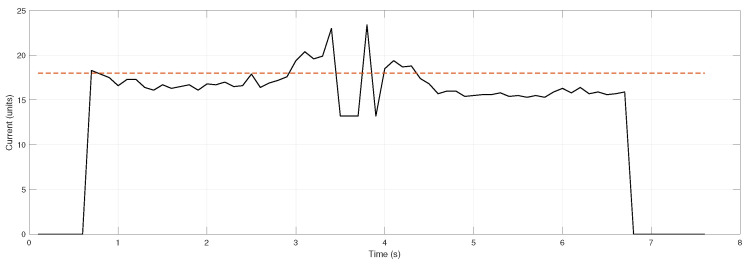
Current measurements and threshold (red dashed line) for the test showed in Figure 5.

When stalling is detected during testing, the motor temporarily halts before resuming motion. Current exceeding the calibrated threshold (red dashed line) triggers a motor stop and the system proceeds with the following steps defined for regular operation. Considering the trial depicted in Figure 5, corresponding the current measurement results are shown in Figure 7.

Current measurements demonstrate proper system operation, with the microcontroller accurately detecting full gripper closure through characteristic current peaks. When measured current surpasses the predefined threshold, the gripper arms are confirmed to be in contact with either the target parcel or the opposing jaw.

**Figure 8:**
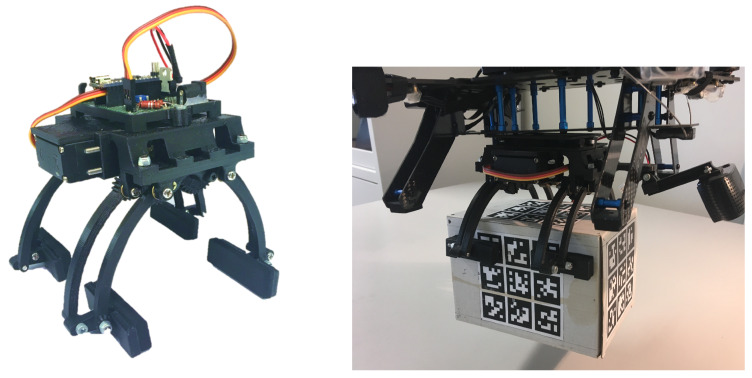
Final gripper prototype as well as the integration with the drone and grasping a parcel.

The final gripper prototype depicted in Figure 8, both standalone and integrated in the drone holding a parcel, was also evaluated for its load-bearing capacity during static conditions.

The bulk of its structure and all of the spur gears were 3D printed using Fused Deposition Modeling with a PLA material, weighing 250 g without the camera and 290 g with the used camera. Experimental tests were performed using a spring-scale dynamometer attached to the bottom surface of a test parcel box to assess the maximum weight that the gripper could hold without electric current being supplied to the motor. It was experimentally observed that the gripper mechanism equipped with the worm gear and end parts fitted with rubber mats could hold cargo of up to 1 kg.

In Table 1, a comparison between the proposed gripper and some recent works found in the literature is presented.

Although its purpose is more specific to the application and usage detailed above, it can be seen that it is lighter than most of the rigid grippers presented in [10,11], which usually weigh more than 300 g, while still being able to handle parcels up to 1 kg, which is above the required payload for the intended application and for the payload capacity for which most small drones are designed to carry. Another design option related to the autonomy of the drone+gripper system is the use of the worm gear, which provides self-locking capabilities, avoiding the need for continuous power supply to the motor to keep the parcel grasped during transportation. This type of discussion is seldomly found in the literature, but it is an important aspect to consider when designing aerial manipulation systems, as it can significantly impact the overall energy consumption and flight endurance of the drone.

## 3. Parcel Pose Estimation

**Figure 9:**
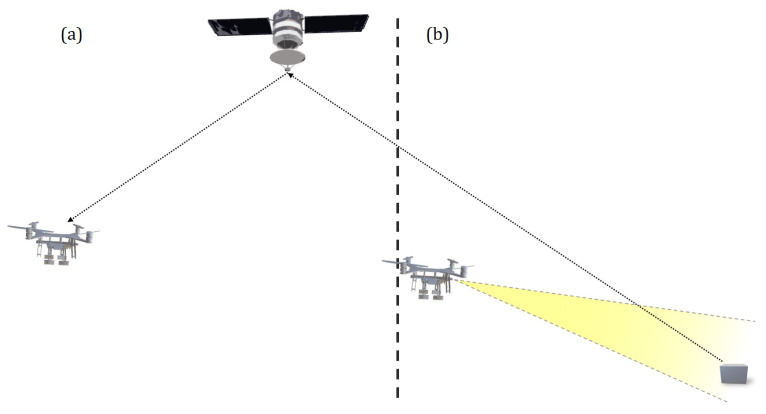
Combination of offboard (**a**) and onboard (**b**) position acquisition.

Accurate determination of the target package position can be achieved through various approaches. External positioning systems, such as GPS tracking, represent one possibility, but their positional uncertainties and limited update frequencies render them unsuitable for close-range operations and rapid maneuvers. Conversely, onboard sensing methods, including computer vision or proximity sensors, typically deliver improved accuracy at shorter ranges with fewer environmental obstructions. The adopted approach combines both methodologies: initial acquisition at larger relative distances with relaxed accuracy requirements, transitioning to precise measurement as separation decreases, corresponding to scenarios (a) and (b) depicted in Figure 9, respectively.

The present work concentrates exclusively on the onboard sensing phase (b), where a camera establishes correspondences between environmental features and their image plane projections. Incorporating passive markers on the package surface significantly enhances both image capture and processing performance by supplying the detection algorithm with predefined reference points. This approach, termed a fiducial marker system, enables parcel pose estimation relative to a monocular camera with low computational cost, substantial robustness, and rapid processing.

### 3.1. ArUco Marker System

Fiducial marker systems operate using predefined marker patterns and algorithms that execute detection, error correction, and pose estimation. Among the various implementations available, refs. [14,28] present a computationally efficient and robust square fiducial marker approach employing binary encoding, with capabilities for detecting and estimating poses of individual markers or marker arrays. The *ArUco* library provides an open-source implementation of this methodology, where the marker generation occurs offline through an optimization algorithm that maximizes inter-marker distance and bit transition count, with each marker assigned a unique identifier and stored in a dictionary. Marker detection within images is executed through the *ArUco* library function detectMarkers().

*ArUco* tags can be deployed either individually or in collective arrangements, where the latter may be distributed across planar surfaces (termed boards) or three-dimensional structures. Three-dimensional marker arrangements prove particularly suitable for package applications, as they provide redundancy to compensate for marker occlusion or partial visibility, enabling detection from arbitrary viewing angles. Constructing a 3D marker structure requires specifying the spatial coordinates of each marker corner, assigning individual marker identifiers, and selecting the appropriate dictionary. Once these parameters are defined, the *ArUco* library function Board_create() generates the corresponding 3D structure object.

**Figure 10:**
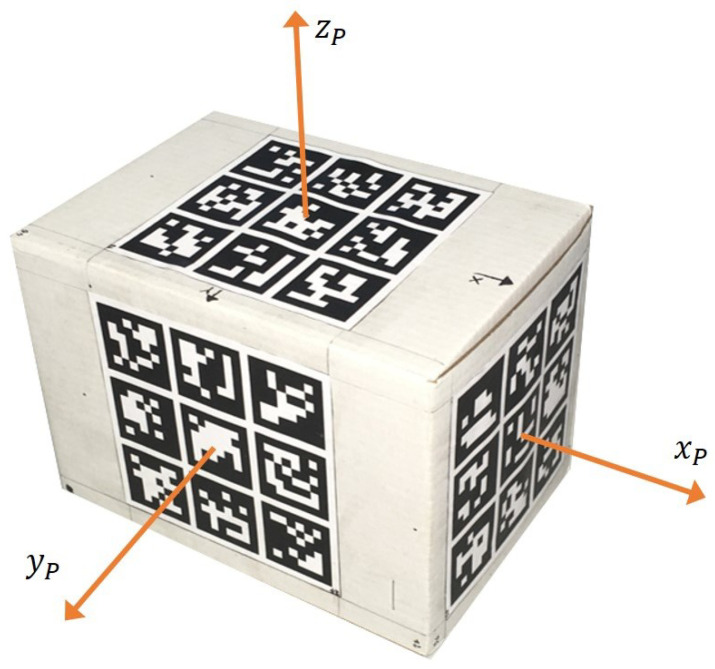
*ArUco* 3D structure with P coordinate frame.

The package reference frame P is established based on the marker configuration geometry, as illustrated in Figure 10, with its origin located at the package centroid and axes $x_{P}$, $y_{P}$ and $z_{P}$ aligned with the box length, width, and height, respectively.

Determining the pose of an *ArUco* marker structure requires the camera’s intrinsic matrix $C_{I}$ and distortion coefficient vector $D_{cf}$, which are camera-specific and obtained through calibration procedures. Providing these camera parameters together with the corner coordinates of each of the $N_{d}$ detected markers, their corresponding *ids*, and the predefined 3D marker geometry to the *ArUco* library function estimatePoseBoard() yields the package pose relative to the camera parameterized by a vector $r_{v}\in R^{3}$ representing the rotation and a vector $t_{v}\in R^{3}$ the translation, where the latter corresponds to the position of P expressed in C, denoted by pPC.

The vector $r_{v}$ representing the rotation can be converted to the rotation matrix representing the relative attitude of P as seen by C, via the *Rodrigues’* rotation formula

(9) RPC=I+S(r¯v)sin(α)+S2(r¯v)(1−cos(α))

where the rotation angle is $\alpha =∥r_{v}∥$, the rotation axis is ${\bar{r}}_{v}=r_{v}/∥r_{v}∥$, *I* represents the 3×3 identity matrix, and S(a) denotes the skew-symmetric matrix that defines the cross product as S(a)b=a×b, where $a,b\in R^{3}$.

### 3.2. Drone-with-Gripper Perception of Package Pose

The camera frame C is mounted beneath the vehicle frame B to maximize parcel visibility while minimizing obstructions during grasping operations. Specifically, C is offset from the B origin by a fixed displacement $l_{o}=[l_{o}^{x},l_{o}^{y},0]$ and rotated about the $y_{B}$ axis by angle $\theta_{o}$. The transformation relating the camera frame C to the vehicle body frame B is then expressed as

(10) RCB=cos(θo)0−sin(θo)010sin(θo)0−cos(θo),

with pCB=−lo. As such, the complete transformation from P to W is given by

(11) pPW=pBW−RBW(pCB+RCBpPC),

considering the parcel orientation in frame W given by RPW=RBWRCBRPC.

### 3.3. ArUco Pose Estimation Evaluation

To evaluate the quality of the discussed method and its applicability in our proposed scenario, a group of tests were made, resembling the expected working conditions. The camera used for these tests was a C290 (by Logitech International S.A., Lausanne, Switzerland) with a stated image resolution of 800 × 600. The sample rate of the pose estimation algorithm is strongly dependent on the camera frame rate and on the computer processing capabilities, which in this case were both able to properly function at 30 Hz.

**Figure 11:**
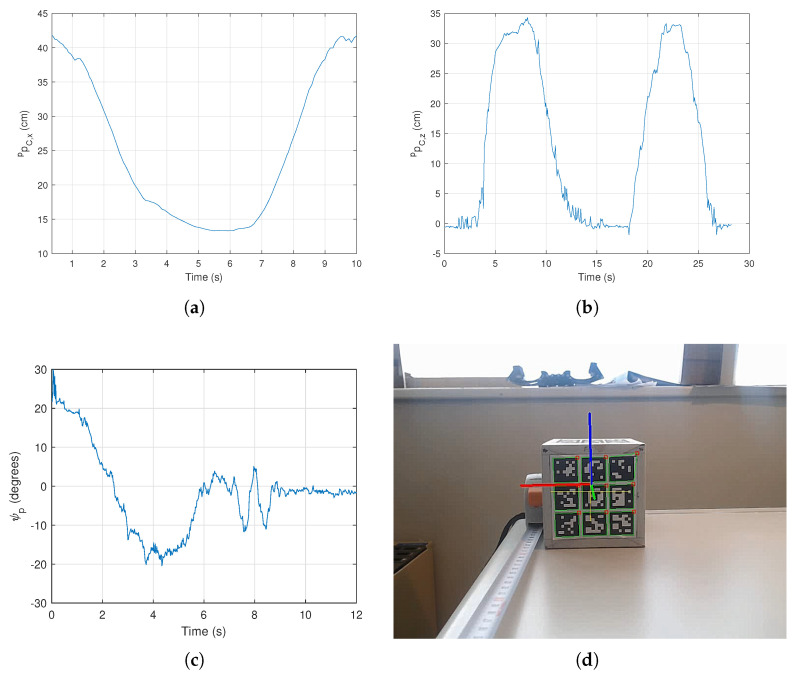
Pose estimation tests: (**a**) *x* axis approach; (**b**) *z* axis approach; (**c**) yaw rotation; (**d**) testing environment.

To better assess algorithm performance and interpret results, outputs are presented as camera pose relative to the parcel frame, pCP. Assuming horizontal parcel placement, P coincides with W, allowing direct interpretation as ground distances. A first test involved horizontal approach along the *x* axis, a second examined *z* axis motion, where non-perpendicular observation increases motion blur susceptibility, and a third test the rotation about the *z* axis was tested. These tests are depicted in Figure 11, along with a picture of the testing environment.

While no groundthrough was available, qualitative assessment confirms adequate accuracy of the pose estimation strategy with minimal noise, attributed to the perpendicular observation angle reducing motion blur. It is also noticeable that *z* axis motion reveals increased noise, though remaining acceptable at approximately 1 cm magnitude, which can be mitigated throught simple filtering techniques.

## 4. Hybrid Grasping Model Predictive Control

**Figure 12:**
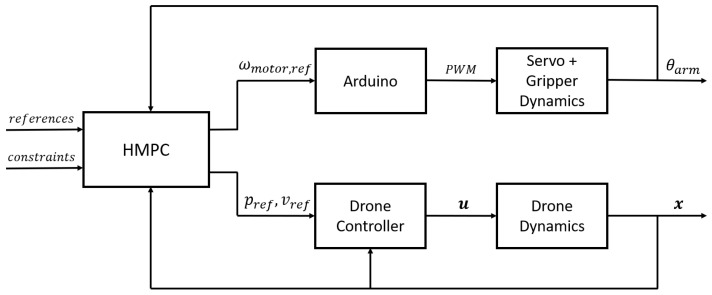
Control loop including the hybrid MPC combining the drone and gripper dynamics.

This section develops dynamic models for both the vehicle and gripper subsystems, subsequently integrating them into a unified hybrid model capable of representing multiple operational scenarios and modes. The objective is to formulate a Hybrid Model Predictive Controller (HMPC) that enforces all critical constraints with minimal deviation. Figure 12 illustrates the control architecture employed in this work, wherein the HMPC computes reference signals for both the gripper and vehicle controllers.

### 4.1. Drone Dynamics

Developing a fully autonomous grasping system for aerial parcel delivery necessitates establishing appropriate quadrotor dynamics and control models, for which the approach followed in [29] is adopted. The rotation matrix of the vehicle body frame B relative to the world frame W, denoted as RBW or simply as *R*, can be parameterized using, for instance, the ZYX Euler angles ϕ (roll), θ (pitch), and ψ (yaw), respectively. This rotation matrix can also be recovered by composing three simple rotations based on the Euler angles, according to the ZYX sequence. The angular velocity of B relative to W expressed in B is denoted as $\omega \in R^{3}$, which can also be related to the time derivatives of Euler angles through an appropriate transformation. Additionally, the position of the origin of B relative to W is denoted by $p\in R^{3}$, whereas its linear velocity is $v\in R^{3}$. Thus, the kinematics and dynamic differential equations that describe the motion of the vehicle can be written as

(12) $$\begin{array}{cc} \dot{p} & =v \end{array}$$

(13) $$\begin{array}{cc} \dot{v} & =-gz_{W}+\frac{u_{1}}{m}z_{B} \end{array}$$

(14) $$\begin{array}{cc} \dot{R} & =RS{\left(\omega \right)}_{B} \end{array}$$

(15) $$\begin{array}{cc} \dot{\omega } & =J^{-1}\left(-S\left(\omega \right)J\omega +u_{\tau }\right) \end{array}$$

where *m* is the mass of the vehicle, *g* is the gravitational acceleration, *J* is the inertia matrix of the vehicle, $u_{1}$ is the total thrust generated by the rotors, and $u_{\tau }={\left[u_{2}\;u_{3}\;u_{4}\right]}^{T}$ is the vector of moments applied to the vehicle in its body frame. Also, vectors $z_{w}$ and $z_{B}$ are the *z* axis in W and B, respectively. These two vectors are related by $z_{B}=Rz_{w}$. An appropriate control law, capable of providing the motor input vector ***u*** that is able to follow a desired trajectory based on position, velocity, and orientation references is thoroughly described in [29].

Considering a hierarchical control strategy, a drone might use several control loops that account, progressively, local control laws for angular velocity, attitude, linear velocity, and position. As typical autopilots provide such inner-loop control laws, a high-level controller such as the one considered here can assume that, if a velocity reference is computed, the autopilot inner loops can easily follow that reference. Thus, the high-level drone model used for integrated guidance and control can be greatly simplified, simply considering $\dot{p}=v$ and $\dot{\psi }=\omega_{z}$, where the inputs are now considered to be the drone linear velocity, v, and the angular rate about the *z* axis, $\omega_{z}$. A discrete-time version of this model can also be defined, considering normalized velocity and yaw-rate inputs, $u_{v}\in {\left[-1,1\right]}^{3}$ and $u_{\psi }\in \left[-1,1\right]$, respectively, as well as one sample time delay, $T_{s}$, on the velocity input, yielding

(16) $$\begin{array}{cc} p(k+1) & =p\left(k\right)+T_{s}C_{v}u_{v}\left(k-1\right) \end{array}$$

(17) $$\begin{array}{cc} \psi (k+1) & =\psi \left(k\right)+T_{s}C_{\psi }u_{\psi }\left(k\right) \end{array}$$

where $C_{v}$ and $C_{\psi }$ are constant parameters. Considering the drone state vector $x_{d}\left(k\right)={\left[p\left(k\right)\;u_{v}\left(k-1\right)\;\psi \left(k\right)\right]}^{T}$ and respective input vector $u_{d}\left(k\right)={\left[u_{v}\left(k\right)\;u_{\psi }\left(k\right)\right]}^{T}$, this model can be rewritten as

(18) $$\begin{array}{cc} x_{d}\left(k+1\right) & =A_{d}x_{d}\left(k\right)+B_{d}u_{d}\left(k\right) \end{array}$$

where

(19) $$\begin{array}{cccc} A_{d} & =\left[\begin{array}{ccc} I & T_{s}C_{v}I & 0_{3\times 1}\\ 0_{3\times 3} & 0_{3\times 3} & 0_{3\times 1}\\ 0_{1\times 3} & 0_{1\times 3} & 1 \end{array}\right] & B_{d} & =\left[\begin{array}{cc} 0_{3\times 3} & 0_{3\times 1}\\ I & 0_{3\times 1}\\ 0_{1\times 3} & T_{s}C_{\psi } \end{array}\right] \end{array}$$

### 4.2. Dynamic Model of the Gripper

The only actuator in the gripper system is a servo motor modified to be able to have an infinite rotation span, controlled by an Arduino microcontroller. This modification removes the original position feedback capability of a servo motor, but enables its position control.

To model the motor dynamics, simple identification tools where used and a 2nd-order discrete-time system is found to be sufficiently accurate, relating the motor angular velocity $\omega_{m}\in R$ with a normalized input $u_{m}\in \left[-1,1\right]$, yielding

(20) $$\omega_{m}\left(k+1\right)=-a_{1}\omega_{m}\left(k\right)-a_{2}\omega_{m}\left(k-1\right)+b_{1}u_{m}\left(k\right)+b_{2}u_{m}\left(k-1\right)$$

where $a_{1}$, $a_{2}$, $b_{1}$, and $b_{2}$ are constant model coefficients. The gripper arm angular velocity is defined as $\omega =S_{total}\omega_{m}$, where $S_{total}$ is the combined gear ratio from the motor to the gripper arm, and the angular position of the arm can also be defined as $\theta =S_{total}\theta_{m}$, where $\theta_{m}$ is the angular position of the motor. Thus, considering a sample time $T_{s}$, $A_{i}=S_{total}a_{i}$, and $B_{i}=S_{total}b_{i}$, the discrete-time dynamics of the gripper can be defined as

(21) $$\begin{array}{cc} \theta (k+1) & =\theta \left(k\right)+T_{s}\omega \left(k\right) \end{array}$$

(22) $$\begin{array}{cc} \omega (k+1) & =-A_{1}\omega \left(k\right)-A_{2}\omega \left(k-1\right)+B_{1}u_{m}\left(k\right)+B_{2}u_{m}\left(k-1\right) \end{array}$$

Considering the gripper state vector $x_{g}\left(k\right)={\left[\theta \left(k\right)\;\omega \left(k\right)\;\omega \left(k-1\right)\;u_{m}\left(k-1\right)\right]}^{T}$, this model can be rewritten as

(23) $$\begin{array}{cc} x_{g}\left(k+1\right) & =A_{g}x_{g}\left(k\right)+B_{g}u_{m}\left(k\right) \end{array}$$

where

(24) $$\begin{array}{cccc} A_{g} & =\left[\begin{array}{cccc} 1 & T_{s} & 0 & 0\\ 0 & -A_{1} & -A_{2} & B_{2}\\ 0 & 1 & 0 & 0\\ 0 & 0 & 0 & 0 \end{array}\right] & B_{g} & =\left[\begin{array}{c} 0\\ B_{1}\\ 0\\ 1 \end{array}\right] \end{array}$$

**Figure 13:**
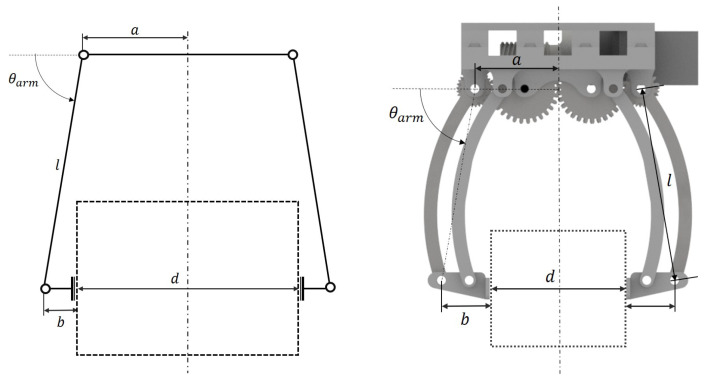
Closed gripper diagram and 3D rendering.

Based on the geometry illustrated in Figure 13, the jaw separation resulting from motor rotation is given by d=2(a+lcos(θ)−b), where *l* represents the gripper arm length, while *a* and *b* denote geometric parameters defining the spacing between gripper arm rotation axes.

Consequently, for a parcel of width $d_{ref}$, the required gripper arm angle is

(25) $$\theta_{ref}=\arccos\left(\frac{\frac{d_{ref}}{2}-a+b}{l}\right).$$

The actual limitations of the arms angle is constrained by the geometry of the parts, which considering a zero parcel dimension, yields $\theta_{max}=\arccos\left(\left(-a+b\right)/l\right)$, whereas $\theta_{min}=0$ due to mechanical limitations.

### 4.3. Hybrid Model

Given that the system does not consist only of state and input variables representing physical quantities, but also on parts described by logic and discrete evolution, a hybrid model can be formulated. To this end, additional variables are defined in order to better model the system, using a notation where binary variables are represented by a $\delta_{var}\in {\left\{0,1\right\}}^{n_{var}}$ and continuous variables by a $\gamma_{var}\in Rn_{var}$. The hybrid model of the drone and gripper system, based on the models introduced above, is described by a set of continuous state variables: θ,ω∈R representing the angle and angular velocity of the gripper arm; $p\in R^{3}$ and $v\in R^{3}$ denoting the drone’s position and velocity in W, and ψ∈R denoting the drone’s yaw angle. Another important state will be the phase in which the hybrid model of the gripper is, which we can enumerate as {A,B,C}, characterizing the current mode of operation. To represent this, a binary vector can be used, such as $\delta_{phase}={\left[\delta_{A}\;\delta_{B}\;\delta_{C}\right]}^{T}\in {\left\{0,1\right\}}^{3}$.

Considering first the continuous state variables and their respective equations defined in (18) and (23), defining the continuous state vector $x_{c}\left(k\right)={\left[x_{g}{\left(k\right)}^{T}\;x_{d}{\left(k\right)}^{T}\right]}^{T}$ and respective input vector $u\left(k\right)={\left[u_{m}\left(k\right)\;u_{d}{\left(k\right)}^{T}\right]}^{T}$, the following state equation defines the discrete-time drone and gripper continuous dynamics:

(26) $$\begin{array}{cc} x_{c}\left(k+1\right) & =A_{c}x_{c}\left(k\right)+B_{c}u\left(k\right) \end{array}$$

where

(27) $$\begin{array}{cccc} A_{c} & =\left[\begin{array}{cc} A_{g} & 0_{4\times 7}\\ 0_{7\times 3} & A_{d} \end{array}\right] & B_{c} & =\left[\begin{array}{cc} B_{g} & 0_{4\times 4}\\ 0_{4\times 1} & B_{d} \end{array}\right] \end{array}$$

**Figure 14:**
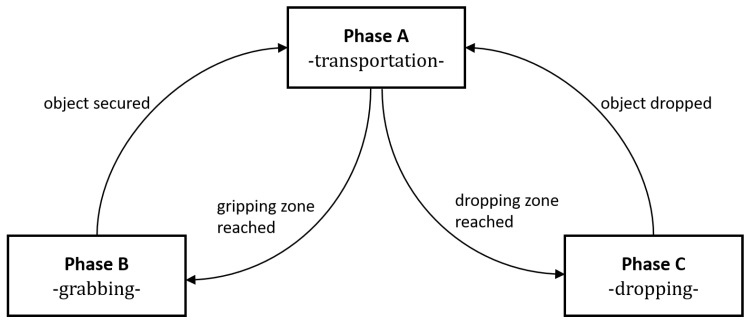
Diagram of the different stages of the hybrid model.

The evolution of the variable $\delta_{phase}$ can be described by the diagram in Figure 14.

Three operational phases are defined to capture both discrete state transitions and continuous dynamics variations within (26). Phase *A* represents conditions where only the drone motion is affected by the controller while the gripper remains inactive, either fully closed or fully opened, which encompasses approach and transport operations, during which the vehicle navigates toward a designated target location. Phase *B* spans the interval from initiation of the grasping maneuver until secure package capture is achieved at the pickup location $p_{grab}\in R^{3}$, where both the drone motion and the gripper are actively controlled. Phase *C* governs package release operations at the delivery location $p_{drop}\in R^{3}$, which also implies the control of both gripper and drone motions. Transitions from phase *A* into phases *B* and *C* occurs upon entering proximity zones around the respective target locations, characterized by $∥p_{a}-p∥\leq d_{zone}$, where $p_{a}$ is either $p_{grab}$ or $p_{drop}$ and $d_{zone}$ is a constant parameter. To each stage corresponds a binary variable, $\delta_{A}$, $\delta_{B}$, or $\delta_{C}$, constrained by $\delta_{A}+\delta_{B}+\delta_{C}=1$, meaning that at any point in time, the system can only be in one of the phases. Concerning the gripper arm rotation span, an auxiliary variable $\delta_{close}\in \left\{0,1\right\}$, specifying when the gripper jaws are fully closed, is created. It is defined as

(28) $$\delta_{close}=\left\{\begin{array}{cc} 0, & \mathrm{if}\theta <\theta_{close}\\ 1, & \mathrm{if}\theta \geq \theta_{close} \end{array}\right.$$

where $\theta_{close}$ is a predefined angle that varies according to the box’s dimensions and can be calculated from (25). An additional binary variable $\delta_{force}$ conveys information about reaction forces applied to the gripper arms, indicating whether cargo is actively being held in these operational phases. The gripper state $\delta_{gripper}$, indicating successful parcel acquisition, is determined through logical combinations of these variables and their complements (denoted by the ¬ operator), expressed as

(29) $$\delta_{gripper}=\left\{\begin{array}{cc} 0, & \mathrm{if}\neg \delta_{force}\wedge \delta_{open}\\ 1, & \mathrm{if}\delta_{force}\wedge \delta_{close} \end{array}\right..$$

Upon successful object capture, the system must immediately transition back to phase *A*, whereas an identical transition occurs following gripper opening. These constraints are expressed as

(30) $$\delta_{gripper}\left(k\right)\wedge \delta_{B}\left(k\right)\Rightarrow \delta_{A}\left(k+1\right),$$

(31) $$\neg \delta_{gripper}\left(k\right)\wedge \delta_{C}\left(k\right)\Rightarrow \delta_{A}\left(k+1\right).$$

A final binary auxiliary variable is necessary to indicate if the drone has reached passed its target location, denoted as $\delta_{passed}$.

With state and auxiliary variables established, the hybrid model can be formulated as a Discrete Hybrid Automaton (DHA). Expressing the model as a DHA enhances comprehension and provides rigorous formalization of the relationships among dynamics and constraints. This formulation further enables systematic conversion to alternative representations, such as Mixed Logical Dynamical (MLD) systems, which characterize the system through linear difference equations incorporating both continuous and binary variables alongside linear inequality constraints, making it well-suited for hybrid model representation and optimization-based control implementations. The HYbrid System DEscription Language (HYSDEL), introduced in [30], provides a modeling framework for specifying DHA models, whereas the methodology for converting to MLD systems and associated language constructs is detailed in [24].

### 4.4. Hybrid Model Predictive Controller

Satisfying the hybrid model’s requirements, constraints, and objectives is most effectively accomplished through Model Predictive Control (MPC), wherein finite-horizon optimal control problems are solved iteratively at each time step. Selecting the prediction horizon, $N_{p}$, necessitates understanding the system’s dominant dynamical behavior, as the controller must anticipate critical operational events with sufficient foresight to enable appropriate corrective actions. Idealy, $N_{p}$ should be greater than the number of time steps necessary to fully close the gripper mechanism to its gripping angle $\theta_{close}$. To prevent the need to use exceedingly large values for $N_{p}$, the gripping strategy consists of first moving the gripper to an intermediate closing angle $\theta_{pre}$, after it reaches a predefined safety zone. This way, the final gripping maneuver requires a considerably smaller prediction horizon and, depending on the choice of parameters, for $T_{s}=0.1$ s, the most efficient prediction horizon is between 5 and 7 time intervals.

The optimization problem to be solved in each iteration of the MPC is formulated as the Mixed Integer Quadratic Programing (MIQP) problem:

(32) $$\begin{array}{cccc} \; & \min_{q_{0}} & J_{0}\left(x\left(k\right),q_{0}\right)\\ \; & \;s.t. & x(k+1) & =\;Ax\left(k\right)+B_{1}u\left(k\right)+B_{2}\delta \left(k\right)+B_{3}z\left(k\right)+B_{5},\\ \; & \; & y(k) & =\;Cx\left(k\right)+D_{1}u\left(k\right)+D_{2}\delta \left(k\right)+D_{3}z\left(k\right)+D_{5},\\ \; & \; & E_{2}\delta \left(k\right)+E_{3}z\left(k\right) & \leq \;E_{1}u\left(k\right)+E_{4}x\left(k\right)+E_{5} \end{array}$$

where the cost function $J_{0}$ is given by

(33) $$J_{0}\left(x\left(k\right),q_{0}\right)={∥x\left(N\right)∥}_{Q_{x_{N}}}^{2}+\sum_{k=0}^{N-1}{[∥y\left(k\right)∥}_{Q_{y}}^{2}+{∥u\left(k\right)∥}_{Q_{u}}^{2}+{∥z\left(k\right)∥}_{Q_{z}}^{2}+{∥x\left(k\right)∥}_{Q_{x}}^{2}]$$

with ${∥x∥}_{Q}^{2}=x^{T}Qx$, $q_{0}$ denoting the vector consisting of the optimization slack variables, the sequence of future values of the inputs *u*, and the auxiliary and output variables δ, *z*, and *y*. The cost functional matrix weights, $Q_{x}$, $Q_{y}$, $Q_{u}$, $Q_{z}$, and $Q_{x_{N}}$, can be chosen according to the relative weights of the current state of the system *x*, the outputs *y*, the auxiliary variables *z*, and the state vector at the prediction horizon $x_{N}$, respectively. The Hybrid Toolbox for MATLAB, presented in [31], provides a mechanism to convert the formulation expressed before into a more desirable compact form, accepted by the most common solvers, such as IBM’s CPLEX or MATLAB’s optimization toolbox.

The $Q_{y}$ matrix takes advantage of a combination of auxiliary variables to compute a more reliable functional cost. First, it is necessary to define the y(k) vector containing the auxiliary variables. Since there are different objectives for each stage, some auxiliary variables are only relevant when the system is at a specific phase. The notation $\gamma_{x}^{ph}\backslash \delta_{x}^{ph}$ is hereby equivalent to $\gamma_{x}\backslash \delta_{x}\wedge phase=ph$. The auxiliary variables used to computed the cost of the optimization problem are

(34) $$\begin{array}{c} y\left(k\right)={\left[\begin{array}{ccccc} \delta_{\neg close\&passed}^{B}\left(k\right) & \gamma_{close}^{B}\left(k\right) & \gamma_{zone}^{B}\left(k\right) & \delta_{motion}^{B}\left(k\right) & \gamma_{drop}^{C}\left(k\right) \end{array}\right]}^{T}. \end{array}$$

Several auxiliary variables contribute to the cost function formulation. The variable $\gamma_{close}$ yields the remaining distance to the target when the gripper has achieved full closure, while $\delta_{\neg close\&passed}$ equals unity when the predicted trajectory includes instances where the vehicle has overshot the target without completing gripper closure. The servo motor command $u_{m}$ is provided by $\gamma_{zone}$ when the vehicle remains beyond a designated safety radius from the target location. Additionally, $\delta_{motion}$ assumes a value of one when the gripper occupies an intermediate configuration rather than a limit position, and $\gamma_{drop}$ supplies the servo motor command only prior to the vehicle reaching $p_{grab}$.

## 5. Experimental Results

This section presents the results obtained from both simulations using the developed gripper prototype integrated with the hybrid MPC framework described in Section 4, as well as experimental trials using the parcel perception system outlined in Section 3.

### 5.1. Hybric MPC Simulation Results

Simulation results were obtained using the hybrid MPC framework described in Section 4, implemented in MATLAB R2018a and using the HYSDEL 3.0 to generate the MIQP problem, which was then solved using IBM CPLEX solver 12.8 (in particular, the solver “cplexmiqp”).

The control parameters were determined through a systematic tuning procedure, initially selecting these based on the system’s physical properties and time constants, the prediction horizon was chosen to balance computational load and performance, whereas the cost function matrices were adjusted iteratively to achieve desired tracking accuracy and control effort trade-offs. The final parameter values are summarized in Table 2.

After defining the model parameters and calibrating the optimization cost weight matrices, some simulation tests were used to assess the overall performance of the system. To provide the hybrid model and MPC with the required information to simulate a scenario with the 3 different phases (altough phase *A* repeats after phases *B* and *C*), it is necessary to define the state references for each phase. Assuming the initial conditions to be $x_{0}={\left[0,0_{1\times 3},p_{ini}^{\prime },0_{1\times 3},0\right]}^{\prime }$, a scenario could be devised where the state references given in each phase are as follows:

- *Phase* $A_{1}$: $x_{ref}={\left[0,0_{1\times 3},p_{grab}^{\prime },0_{1\times 3},\psi_{grab}\right]}^{\prime }$
- *Phase B*: $x_{ref}={\left[\theta_{close},0_{1\times 3},p_{grab},0_{1\times 3},\psi_{grab}\right]}^{\prime }$
- *Phase* $A_{2}$: $x_{ref}={\left[\theta_{close},0_{1\times 3},p_{drop},0_{1\times 3},\psi_{grab}\right]}^{\prime }$
- *Phase C*: $x_{ref}={\left[0,0_{1\times 3},p_{drop},0_{1\times 3},0\right]}^{\prime }$
- *Phase* $A_{3}$: $x_{ref}={\left[0,0_{1\times 3},p_{end},0_{1\times 3},0\right]}^{\prime }$

where $\psi_{grab}$ is the yaw angle of the parcel at the grasping position to be acquired by the camera.

**Figure 15:**
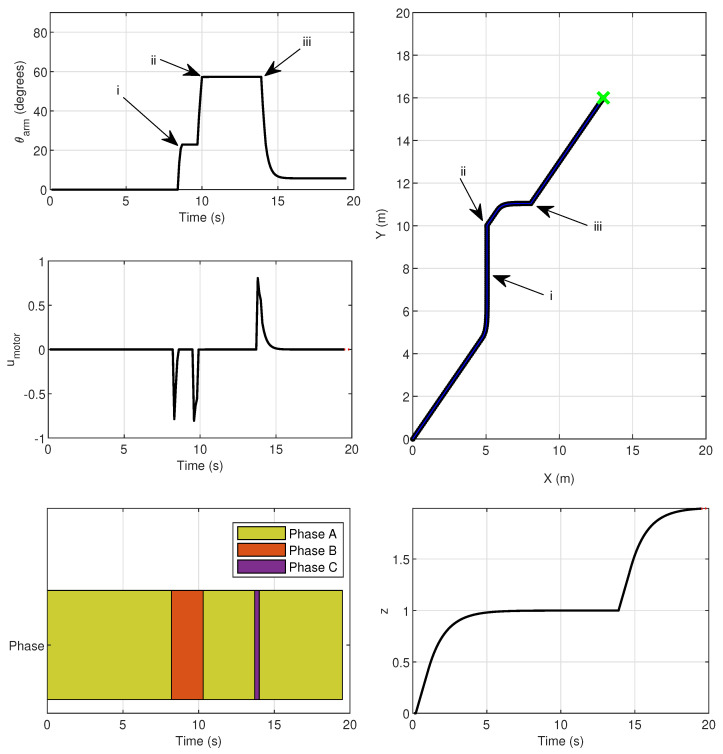
Hybrid MPC simulation. (i) Gripper closes to an intermediate angle $\theta_{pre}$ when the drone reaches the safety zone around $p_{grab}$; (ii) Gripper fully closes when the drone reaches $p_{grab}$; (iii) Gripper opens when the drone reaches $p_{drop}$, (green marker).

Figure 15 shows the results of a simulation using a reference structure as the one described above.

The hybrid MPC outer-loop controller will relay the reference values of ω to the gripper mechanism as well as ***v*** and ψ as desired references to the drone inner-loop controllers.

It is possible to observe that the gripper arm fully closes at the exact moment (ii) then it reaches the desired position $p_{grab}$. It accomplishes this through the strategy described in Section 4.4, where the gripper arm first rotates to $\theta_{pre}$ before fully rotating to $\theta_{close}$. The MPC is able to compute a trajectory that passes through the specified target locations according to the gripper arm angle $\theta_{a}rm$. The dropping of the parcel, represented by the opening of the gripper arm, also occurs at the exact expected moment (iii). These gripper arm movements are enforced by the motor inputs $u_{motor}$ computed with the MPC. It is also confirmed that the hybrid model phases evolve as expected. The system phases transition according to Figure 14. Phase *B*, or the grasping motion sequence, happens between moments (i) and (ii) and is enabled when the drone enter the neighborhood of $p_{grab}$. Phase *C*, or the parcel dropping motion sequence, occurs almost instantaneously after moment (iii), when the drone is at $p_{drop}$. The simulation results indicate that the hybrid MPC is capable of effectively managing the gripper mechanism’s operation in conjunction with the drone’s movement, ensuring precise timing for grasping and releasing the parcel.

### 5.2. Grasping Experimental Results

**Figure 16:**
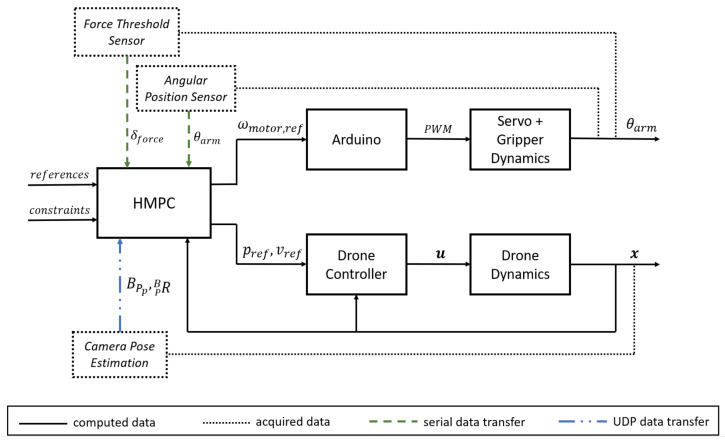
Experimental setup of gripper prototype integrated with the HMPC framework.

For the experimental trials described hereafter, only the gripper mechanism is actuated, considering the simulated drone dynamics using the model described in Section 4.1 while manually moving the gripper system. The experimental setup that includes the gripper hardware and the HMPC is depicted in Figure 16.

The data acquired from the angular position sensor and force threshold sensor was relayed to the main processing unit by an Arduino microcontroller through serial communication at 100 Hz, whereas the camera module provided the parcel position and yaw angle at 30 Hz, relayed to the HMPC solver through a UDP socket. With this information, the HMPC computes at 10 Hz the motor control input $u_{m}$ as well as the desired drone velocities, $v_{r}ef=u_{v}$, and yaw rate, $\omega_{z,ref}=u_{\psi }$, to provide the inner loops for drone motion control and the gripper arm actuation.

**Figure 17:**
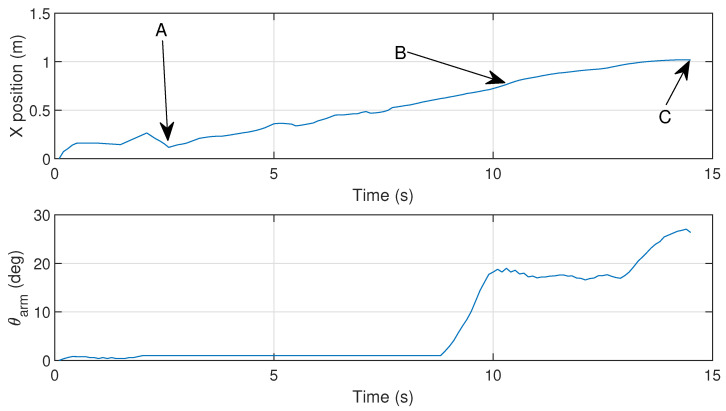
Hybrid MPC experiment: *x* axis position and gripper arm angle, considering the three moments, also depicted below in Figure 19: (A) Approaching the parcel; (B) Pre-closing the gripper arm; (C) Grasping the parcel.

**Figure 18:**
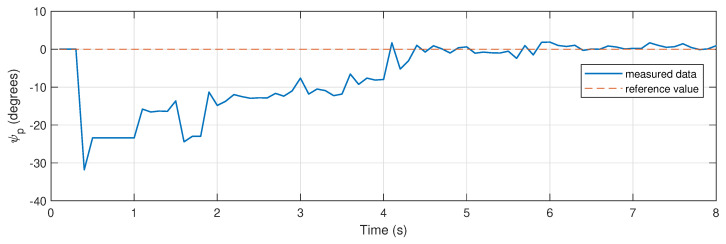
Hybrid MPC experiment: drone–parcel alignment measurements.

Both the yaw angle and position of the parcel are shown in Figure 17 and Figure 18. The drone’s position is given by the distance to the parcel where $p_{grab,x}=1\;m$. For this scenario, the gripper arm angle references are defined by $\theta_{pre}=18^{\circ }$ and $\theta_{close}=28^{\circ }$. This trial is also illustrated in a video (Video S1 in Supplementary Materials).

It is observable that the gripper arm rotates as expected, performing the pre-closing maneuver in order to fully prehend the object in the correct time. The confirmation of a secure parcel prehension comes from the distance computed from the camera in conjunction with the binary variable $\delta_{force}$ obtained from the electric current sensor described above.

Figure 18 shows the yaw angle alignment between the gripper and the parcel during the experimental trial, which is drived towards an acceptable bound during the maneuver, ensuring a successful grasp.

**Figure 19:**
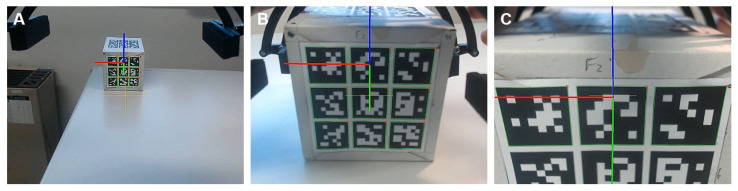
Hybrid MPC experiment: Camera snapshots from different moments during the prehension maneuver: (A) Approaching the parcel; (B) Pre-closing the gripper arm; (C) Grasping the parcel.

Figure 19 presents snapshots taken by the camera module at different moments during the prehension maneuver, also identified as (A, B, C) in Figure 17.

The presented experimental results validate the proposed hybrid MPC framework’s capability to coordinate the gripper mechanism’s operation with the drone’s positioning, ensuring accurate timing for grasping maneuvers based on real-time parcel pose estimation. Nonetheless, it is important to acknowledge that these trials were conducted under controlled conditions, with the drone’s motion simulated and the parcel remaining stationary, and further experimental validation is necessary to assess the system’s performance in dynamic scenarios involving actual drone flight and moving parcels. 

## 6. Conclusions

This paper described the development and experimental assessment of an autonomous grasping system designed for integration with aerial vehicles in parcel delivery and exchange operations. The implementation involved constructing an operational gripper prototype equipped with angular position sensors and force detection capabilities, alongside a pose estimation method for packages, with all components coordinated through a Hybrid Model Predictive Controller that determines optimal vehicle trajectories and gripper actuation commands. Individual subsystems underwent successful testing, and the complete integrated system was validated through both simulated and physical experiments, where package pose was determined via the vision-based estimation algorithm using the onboard camera.

Extensions to agile maneuvers and multi-vehicle scenarios within the hybrid modeling framework represent a potential avenue for further research, as do alternative approaches for hybrid MPC formulation and implementation, given that the HYSDEL formalism has limitations in representing complex nonlinear hybrid dynamics with sufficient fidelity. Additionally, future research could focus on establishing theoretical stability foundations to ensure robust performance across a wider range of operating conditions as well as to use robust MPC techniques for disturbance rejection.

While the presented results provide an initial validation of the proposed approach, further research is necessary to advance toward a fully operational aerial grasping solution. This line of work could conduct extensive experimental trials in real-world scenarios with onboard computation, evaluating the system’s performance under varying environmental conditions and with different parcel types to validate its robustness and adaptability, particularly to moving parcels of increasing agile motions.

## Figures and Tables

**Table 1:** Comparison of the proposed gripper with recent works found in the literature.

Gripper	Weight (kg)	Max. Payload (kg)
T Model [25]	0.4	1.5
Dual Arm [15]	1.8	0.75
Adaptive [26]	0.3	0.06
Compliant [27]	0.009	0.06
Proposed	0.25	1.0

**Table 2:** Hybrid Model simulation parameters.

Parameter	Value
$\theta_{close}$	57.3 deg
$\theta_{pre}$	17.2 deg
$k_{p}$	0.9
$T_{s}$	0.1 s
$N_{p}$	6
$Q_{x}$	diag([30,1,20,1,20,1,10,1,10,0,0])
$Q_{x_{N}}$	diag([30,1,20,1,50,1,10,1,10,0,0])
$Q_{y}$	diag([10,100,1,50,10])
$Q_{u}$	diag([10,1,1,1,1])
$p_{ini}$	${\left[0,0,0\right]}^{\prime }$
$p_{grab}$	${\left[5,10,1\right]}^{\prime }$
$p_{drop}$	${\left[8,11,1\right]}^{\prime }$
$p_{end}$	${\left[13,16,2\right]}^{\prime }$

## Data Availability

The original contributions presented in this study are included in the article/Supplementary Material. Further inquiries can be directed to the corresponding author.

## Supplementary Materials

The following supporting information can be downloaded at https://www.mdpi.com/article/10.3390/s26020653/s1. Video S1: experimental validation of the proposed techniques.
